# Supplementary material for: Rapidly increasing macroalgal cover not related to herbivorous fishes on Mesoamerican reefs
Source: PeerJ. 2016 May 31;4:e2084. doi: 10.7717/peerj.2084 (PMC4893329; doi:10.7717/peerj.2084)
Supplement: Supplemental Information 1 — Site country classification was split into three binary variables. All sites in Northern Quintana Roo (Mexico) are considered to have been impacted by hurricanes in 2005. Some sites were surveyed in August and September 2005 after Hurricane Emily (17/7/2005), although this was not considered to have a sizeable effect on the results as Wilma (21–24/10/2005) was the strongest hurricane registered in the Caribbean (Álvarez-Filip & Gil, 2006). [file peerj-04-2084-s001.docx]

| **Variable** | **Type** | **Description** |
| --- | --- | --- |
| Mexico | Binary | Binary indicator of location of site within Mexico |
| Belize | Binary | Binary indicator of location of site within Belize |
| Honduras | Binary | Binary indicator of location of site within Honduras |
| Island | Binary | Binary indicator of island or continental (mainland) site |
| Hurricane | Binary | Binary indicator of sites affected by 2005 hurricanes |
| Log_10_(Fish_Initial) | Continuous | Logarithm of initial herbivorous fish biomass. Log values used to modify scale |
| Fish_Log_Diff | Continuous | Annual logarithmic change in herbivorous fish biomass |
| Algae_Initial | Continuous | Initial benthic cover by fleshy macroalgae. Decimal values from 0 to 1 representing 0 % to 100 % |
| Algae_Diff | Continuous | Absolute annual percentage change in fleshy macroalgal cover (decimal values) |
| Coral_Initial | Continuous | Initial benthic cover by hard coral. Decimal values from 0 to 1 representing 0 % to 100 % |
| NTZ | Binary | Binary indicator of location of site within a No Take Zone |
| MPA | Binary | Binary indicator of location of site within a Marine Protected Area |
