# Supplementary figures and images for: Rapidly increasing macroalgal cover not related to herbivorous fishes on Mesoamerican reefs
Source: PeerJ. 2016 May 31;4:e2084. doi: 10.7717/peerj.2084 (PMC4893329; doi:10.7717/peerj.2084)

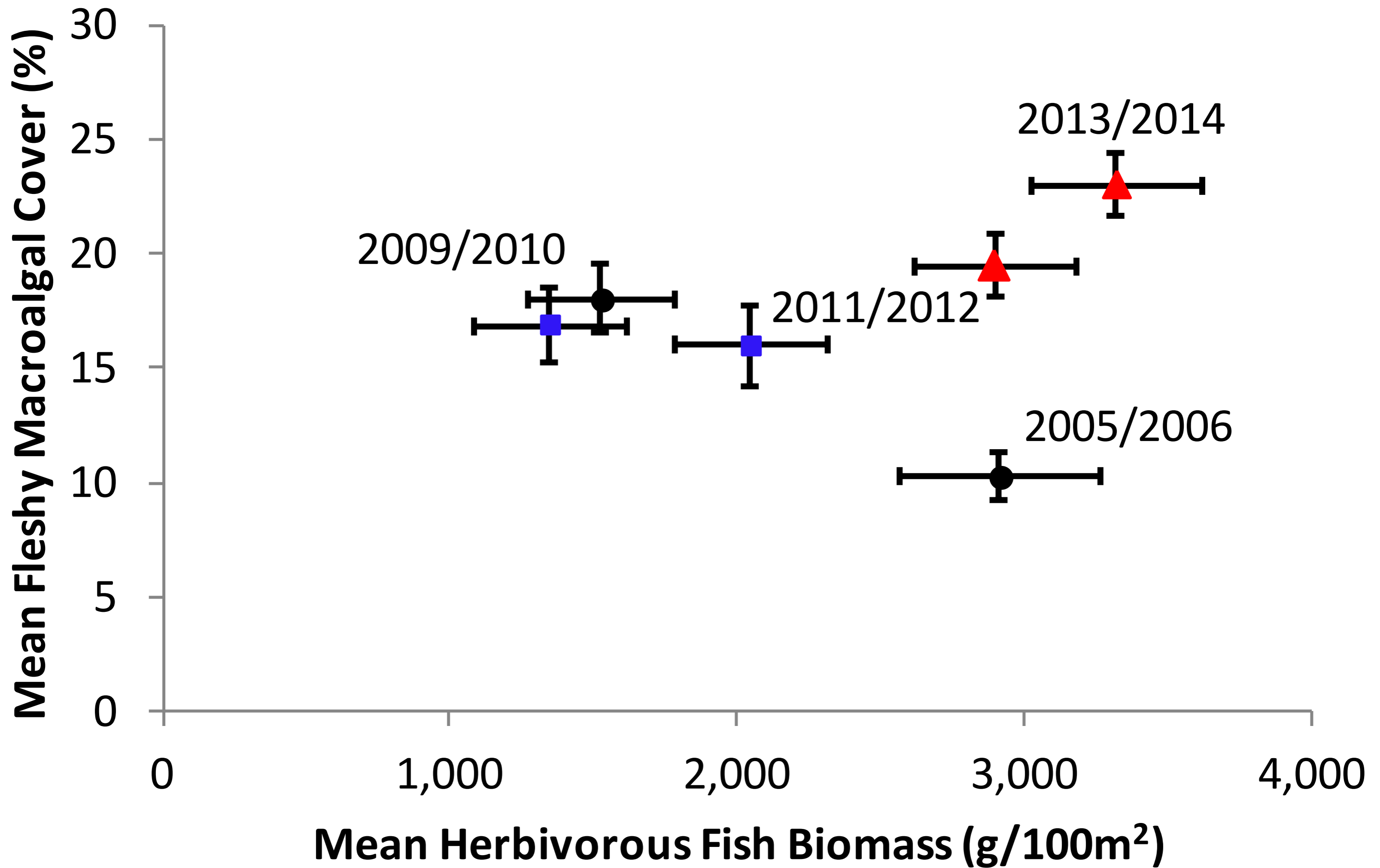

Supplement: Supplemental Information 2 — Mean (± s.e.m.) values are shown for all sites surveyed repeatedly in consecutive monitoring periods. 62 sites were surveyed in both 2005/2006 and 2009/2010 (black circles), 50 sites in 2009/2010 and 2011/2012 (blue squares), and 90 sites in 2011/2012 and 2013/2014 (red triangles). [file peerj-04-2084-s002.pdf]

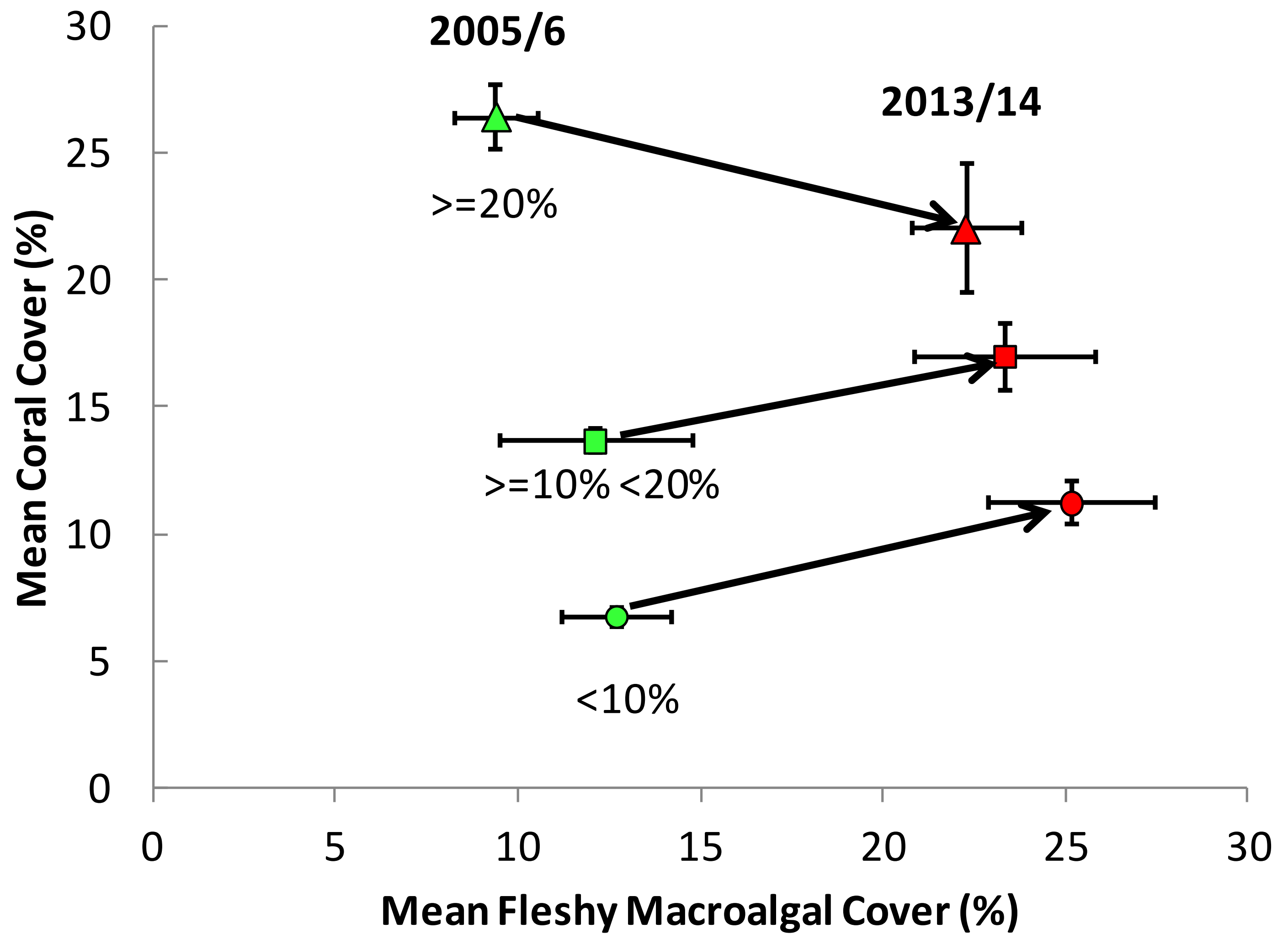

Supplement: Supplemental Information 4 — Mean (± s.e.m.) benthic macroalgal and coral cover for first (2005/6) and last years (2013/14) by initial level of coral cover, for all (85) long-term monitoring sites. Initial level of coral cover categories are < 10% (n = 40); ≥ 10% and < 20% (n = 33); and ≥ 20% (n = 12) benthic cover. [file peerj-04-2084-s004.pdf]

# Herbivorous Fish Community Biomass

## Composition

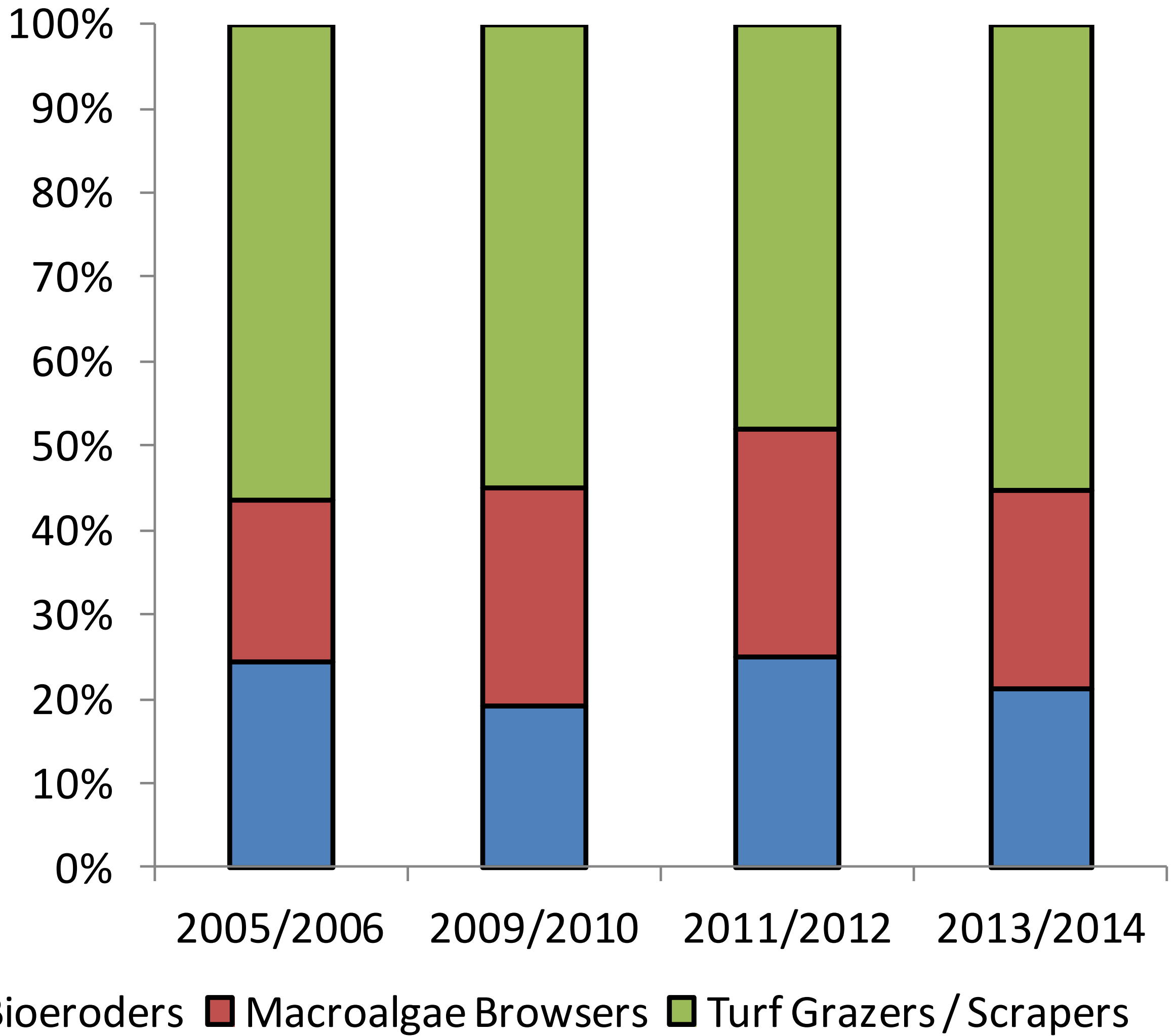

Supplement: Supplemental Information 5 — Percentage composition of herbivorous fish community biomass by feeding guild (bioeroders, macroalgae browsers and turf grazers/scrapers) for sites surveyed repeatedly in each monitoring period (2005/2006, 2009/2010, 2011/2012 and 2013/2014). [file peerj-04-2084-s005.pdf]

High

Mid

Low

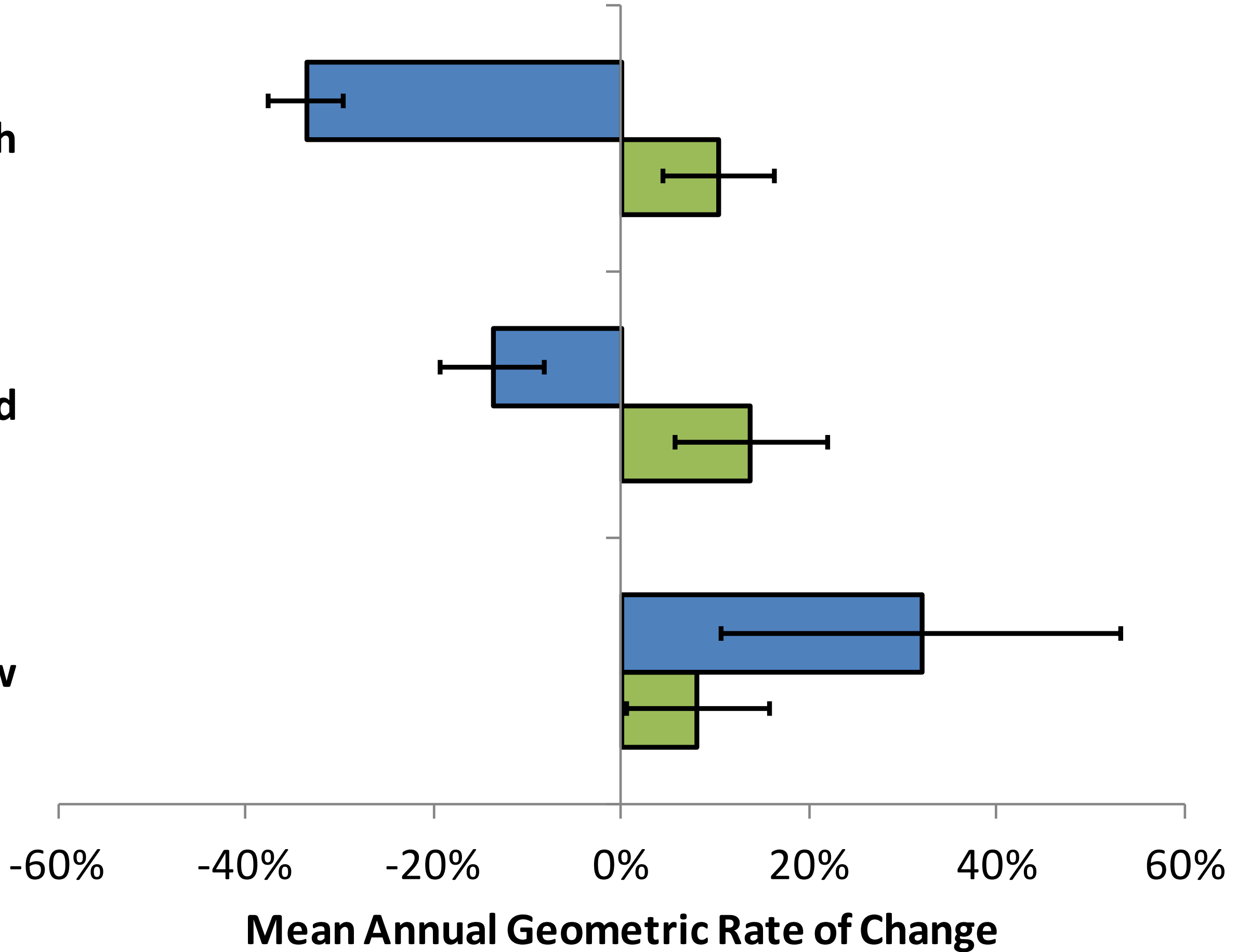

■ Herbivorous Fish 2005-2009 ■ Macroalgal Cover 2009-2014

Supplement: Supplemental Information 6 — Mean (± s.e.m.) annual geometric rates of change for herbivorous fish biomass and fleshy macroalgal cover for sites surveyed repeatedly in each monitoring period (2005/2006, 2009/2010, 2011/2012 and 2013/2014). Sites are grouped into terciles (Low, Mid, High) by initial (2005/2006) herbivorous fish biomass. [file peerj-04-2084-s006.pdf]
