# Supplementary material for: Rapidly increasing macroalgal cover not related to herbivorous fishes on Mesoamerican reefs
Source: PeerJ. 2016 May 31;4:e2084. doi: 10.7717/peerj.2084 (PMC4893329; doi:10.7717/peerj.2084)
Supplement: Supplemental Information 3 — Map and inset graph indicating relationship between changes in herbivorous fish biomass and fleshy macroalgal cover from first (2005 or 2006) to last (2013 or 2014) year for all (73) long-term monitoring sites with ≥ 8 years’ history. For inset graph, each circle represents the sites for that quadrant and circle position reflects mean annual geometric rates of change. Circle area represents proportion of sites in that quadrant (also labelled). [file peerj-04-2084-s003.pdf]

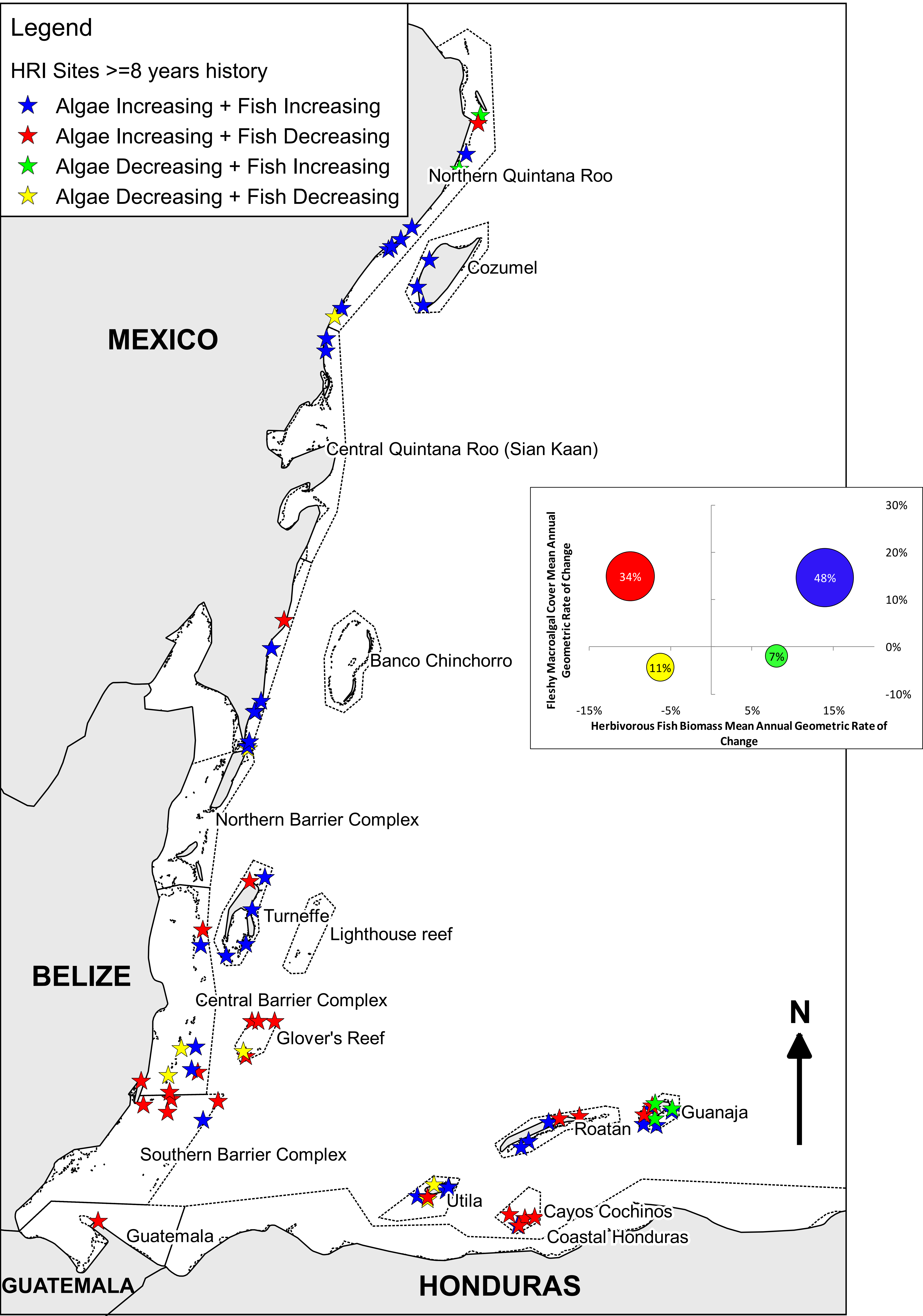
Legend
HRI Sites >=8 years history
Algae Increasing + Fish Increasing
Algae Increasing + Fish Decreasing
Algae Decreasing + Fish Increasing
Algae Decreasing + Fish Decreasing
MEXICO
Northern Quintana Roo
Cozumel
Central Quintana Roo (Sian Kaan)
Banco Chinchorro
Northern Barrier Complex
Turneffe
Lighthouse reef
BELIZE
Central Barrier Complex
Glover's Reef
Southern Barrier Complex
Guatemala
GUATEMALA
HONDURAS
Roatan
Guanaja
Utila
Cayos Cochinos
Coastal Honduras
N
Fleshy Macroalgal Cover Mean Annual Geometric Rate of Change
Herbivorous Fish Biomass Mean Annual Geometric Rate of Change
30%
20%
10%
0%
-10%
-15%
-5%
5%
15%
34%
48%
7%
11%
